# Cannabinoids Drugs and Oral Health—From Recreational Side-Effects to Medicinal Purposes: A Systematic Review

**DOI:** 10.3390/ijms22158329

**Published:** 2021-08-03

**Authors:** Luigi Bellocchio, Alessio Danilo Inchingolo, Angelo Michele Inchingolo, Felice Lorusso, Giuseppina Malcangi, Luigi Santacroce, Antonio Scarano, Ioana Roxana Bordea, Denisa Hazballa, Maria Teresa D’Oria, Ciro Gargiulo Isacco, Ludovica Nucci, Rosario Serpico, Gianluca Martino Tartaglia, Delia Giovanniello, Maria Contaldo, Marco Farronato, Gianna Dipalma, Francesco Inchingolo

**Affiliations:** 1 INSERM, U1215 NeuroCentre Magendie, Endocannabinoids and Neuroadaptation, University of Bordeaux, 33063 Bordeaux, France; 2 Department of Interdisciplinary Medicine, University of Study “Aldo Moro”, Policlinico, 70124 Bari, Italy; ad.inchingolo@libero.it (A.D.I.); angeloinchingolo@gmail.com (A.M.I.); giuseppinamalcangi@libero.it (G.M.); luigi.santacroce@uniba.it (L.S.); denisahazballa@gmail.com (D.H.); mtdoria51@gmail.com (M.T.D.); drciroisacco@gmail.com (C.G.I.); giannadipalma@tiscali.it (G.D.); francesco.inchingolo@uniba.it (F.I.); 3 Department of Medical, Oral and Biotechnological Sciences, University of Chieti-Pescara, 66100 Chieti, Italy; ascarano@unich.it; 4 Department of Oral Rehabilitation, Faculty of Dentistry, Iuliu Hațieganu University of Medicine and Pharmacy, 400012 Cluj-Napoca, Romania; 5 Kongresi Elbasanit, Rruga: Aqif Pasha, 3001 Elbasan, Albania; 6 Department of Medical and Biological Sciences, University of Udine, via delle Scienze, 206, 33100 Udine, Italy; 7 Human Stem Cells Research Center HSC, Ho Chi Minh 70000, Vietnam; 8 Embryology and Regenerative Medicine and Immunology at Pham Chau Trinh, University of Medicine, Hoi An 51300, Vietnam; 9 Multidisciplinary Department of Medical-Surgical and Dental Specialties, University of Campania Luigi Vanvitelli, via Luigi de Crecchio, 680138 Naples, Italy; ludovica.nucci@unicampania.it (L.N.); rosario.serpico@unicampania.it (R.S.); maria.contaldo@unicampania.it (M.C.); 10 UOC Maxillo-Facial Surgery and Dentistry, Department of Biomedical, Surgical and Dental Sciences, School of Dentistry, Fondazione IRCCS Ca Granda, Ospedale Maggiore Policlinico, University of Milan, 20100 Milan, Italy; gianluca.tartaglia@unimi.it (G.M.T.); marco.farronato@unimi.it (M.F.); 11 Hospital A.O.S.G. Moscati, Contrada Amoretta, cap, 83100 Avellino, Italy; giovanniellodelia@gmail.com

**Keywords:** oral health, cannabis, therapeutic adjuvant, mouth diseases

## Abstract

Background: marijuana, the common name for cannabis sativa preparations, is one of the most consumed drug all over the world, both at therapeutical and recreational levels. With the legalization of medical uses of cannabis in many countries, and even its recreational use in most of these, the prevalence of marijuana use has markedly risen over the last decade. At the same time, there is also a higher prevalence in the health concerns related to cannabis use and abuse. Thus, it is mandatory for oral healthcare operators to know and deal with the consequences and effects of cannabis use on oral cavity health. This review will briefly summarize the components of cannabis and the endocannabinoid system, as well as the cellular and molecular mechanisms of biological cannabis action in human cells and biologic activities on tissues. We will also look into oropharyngeal tissue expression of cannabinoid receptors, together with a putative association of cannabis to several oral diseases. Therefore, this review will elaborate the basic biology and physiology of cannabinoids in human oral tissues with the aim of providing a better comprehension of the effects of its use and abuse on oral health, in order to include cannabinoid usage into dental patient health records as well as good medicinal practice. Methods: the paper selection was performed by PubMed/Medline and EMBASE electronic databases, and reported according to the PRISMA guidelines. The scientific products were included for qualitative analysis. Results: the paper search screened a total of 276 papers. After the initial screening and the eligibility assessment, a total of 32 articles were considered for the qualitative analysis. Conclusions: today, cannabis consumption has been correlated to a higher risk of gingival and periodontal disease, oral infection and cancer of the oral cavity, while the physico-chemical activity has not been completely clarified. Further investigations are necessary to evaluate a therapeutic efficacy of this class of drugs for the promising treatment of several different diseases of the salivary glands and oral diseases.

## 1. Introduction

Cannabis, also known as marijuana, has always been one of the illicit drugs most commonly used at recreational levels worldwide [1]. On the other hand, medical use of this plant dates back more than 2000 years ago and has been described in almost all of the ancient cultures [2]. Recreational and ritual use of cannabis and its derived compounds (called cannabinoids) has an important historical meaning, mostly due to the various psychological and physiological effects on the human body, particularly the intense euphoria experience. At the same time, cannabinoids have always been provided to patients, for pain treatment and management, as well as treatment for other types of diseases. Phyto-cannabinoids have been proposed as dietary supplements to improve the gastrointestinal tract function [3,4,5,6]. However, acute and long-term cannabinoid intoxication has several adverse effects which span from unconscious health problems such as tachycardia, immune depression and increased cancer risk [7] to motor impairment and catalepsy [8], interference with cognitive function, panic attacks and a higher risk of developing psychosis [9]. In regard to therapeutic administration, the cannabinoids reported a clinical capability towards anxiety and depressive symptoms regulation [10].

In recent years, many states legalized and promoted the use of cannabinoids for therapeutical purposes, and in some states recreational cannabis became legal and its prevalence markedly rose [11]. Given the present and future increase in health issues related to cannabinoid consumption, it is mandatory for oral healthcare providers and dentists to know and understand the oral effects of cannabis.

This review briefly summarized the components of cannabis and the endocannabinoid system, as well as its cellular and molecular mechanisms of biological cannabis action in human cells and biologic activities on tissues. We will also look into oropharyngeal tissue expression of cannabinoid receptors together with a putative association of cannabis to several oral diseases. Therefore, this review elaborates the basic biology and physiology of cannabinoids in human oral tissues, with the aim of providing a better comprehension of the effects of its use and abuse on oral health, in order to include cannabinoid usage into dental patient health records as well as good medicinal practice.

### 1.1. Cannabinoids and Their Biological Effects

#### 1.1.1. Phyto-Cannabinoids

The *Cannabis sativa* plant contains more than 500 components. Amongst them, more than 100 compounds which possess an aromatic hydrocarbon have been identified and called cannabinoids [12]. All these cannabinoids have bind-described bind/activate cannabinoid receptors [13,14]. The plant-derived cannabinoids are also called phyto-cannabinoids, in order to distinguish them from synthetic cannabinoids and endogenous counterparts (endocannabinoids). Among phyto-cannabinoids there are three major compounds derived from cannabigerol-type (CBG) molecules, delta-9-tetrahydrocannabinol (THC, the main psychoactive compounds from cannabis), cannabinol (CBN), and cannabidiol (CBD) [15]. They were isolated and structurally identified by nuclear magnetic resonance as well as by mass spectrometry [16]. The majority of phyto-cannabinoids are characterized by different affinities to cannabinoid receptors, despite possessing the basic structural types described above.

THC, a highly hydrophobic and lipophilic compound, is the most abundant in cannabis [17]. This compound binds to both cannabinoid receptors with similar affinities for CB_1_ and CB_2_ (both Ki values are around 40 nM), but has been shown to possess less intrinsic affinity to CB_2_ than CB_1_ [14]. THC administration to animal models as well as to human subjects highlighted the enormous and potent psychoactive properties of this compound, with a plethora of effects on locomotion, anxiety, pain, cognition and reality perception [1,18]. On the other hand, CBD has always been considered to be an isomer of THC devoid of psychoactive activity. When compared to THC, CBD has significantly lower affinity for CB_1_ and CB_2_ receptors, with Ki values at M levels (in nM for THC) [14], but several other brain targets and molecular effectors have been proposed for this compound other than cannabinoid receptors, including numerous classical ion channels, receptors, transporters, and enzymes (reviewed in [19]). However, some CBD effects at these targets in in vitro assays only manifest at high concentrations, which may be difficult to achieve in vivo, particularly given CBD’s relatively poor bioavailability [20]. Several reports also suggest that CBD might also affect the bioavailability, receptor binding and molecular actions of THC [21].

CBN is a product of THC metabolism and has only mild psychoactive activity if compared to its parental molecule [22] with higher affinity to CB_2_ than CB_1_ receptors. To date, there are three main forms of cannabis consumption: marijuana, hashish, and hash oil [23]. Hemp, a preparation of cannabis dried leaves and flowers, contains 0.5%–5% THC. On the other hand, cannabis flower heads compressed to form small light brown or black blocks, so called hashish, contains 2%–20% THC. The recently formulated hash oil, which is an oily liquid derived from hashish, can include up to 15%–50% THC and represents the highest percentage obtained in natural products so far [23].

#### 1.1.2. Synthetic Cannabinoids

**Figure 1:**
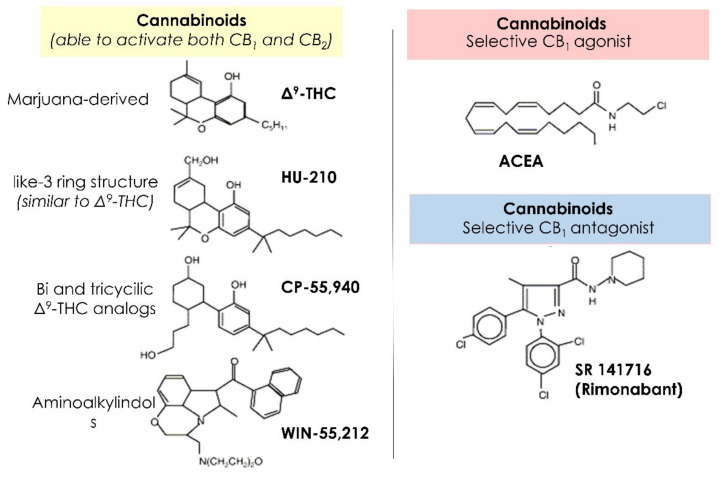
Summary of the main cannabinoids selective for CB_1_ and CB_2_ receptors.

Historically, the use of the marijuana-derived Δ_9_-THC as well as synthetic analogues was actually the golden tool for the discovery and characterization of CB_1_ [24]. Among the synthetic cannabinoid agonists, we will briefly mention some of them, since they are widely used in experimental models (Figure 1). HU-210, characterized by a like-3 ring structure as in THC, is the most potent synthetic compound belonging to the HU series and was first synthesized and characterized in Israel. Bi- and tricyclic analogs of Δ_9_-THC, such as CP-55,940, characterize the second group of CB_1_ agonists used in pharmacological studies. As a third group of ligands, amino-alkylindols, such as WIN-55,212, exhibit potent CB_1_ agonistic activity [12].

All the above reported compounds also show some ability to bind and activate CB_2_ receptors. Amongst the selective CB_1_ agonists, ACEA (arachidonoyl-2′-chloroethanolamide) is the first one ever characterized and has a very potent and extremely selective CB_1_ agonist without activity at CB_2_ [25]. Synthetic ligands showing antagonistic properties at the cannabinoid receptors have been developed in the past. The compounds specific to CB_1_ and most widely used in both pre-clinical and clinical studies are SR141716 [26], AM251 [27] and AM281 [28]. Instead, CB_2_ receptor antagonists such as SR144528 and AM630 have different actions on effector cells and tissues by targeting the receptors [29]. Finally, two classes of compounds are normally used to interfere with the endocannabinoid system, although not acting directly on cannabinoid receptors. These compounds are represented by inhibitors of endocannabinoid re-uptake, such as AM 404 [30], VDM-11, UCM-707 and OMDM-2 [31], and by inhibitors of anandamide hydrolysis, such as URB532, URB597 [32]. More recently, synthetized compounds are the two inhibitors of 2-AG degradation, such as JZL184 and JZL195 [33,34]. These classes of compounds seem to have been shown to selectively increase the concentration of endocannabinoids, possibly avoiding some of the side effects due to generalized cannabinoid receptor activation by direct agonists.

#### 1.1.3. Cannabinoid Receptors

CB_1_, the first identified cannabinoid receptor identified, was cloned in rat, human and mouse tissues [24,35,36]. The characterization and the cloning of the other well-known cannabinoid receptors, designated CB_2_, were subsequently also realized in the three species [37,38].

The analysis of the primary amino acid sequence of CB_1_ and CB_2_ receptors led to assigning them to the large family of G protein-coupled receptors (GPCRs). A combination of mutagenesis experiments and three dimensional models of these two receptors identified important structural determinants of the structure/function relationships and ligand binding/effector triggering (reviewed in [39]). CB_1_ and CB_2_ are encoded by different genes but possess 44% amino acid homology. In humans, CB_1_ was preferentially localized in the brain and the spinal cord but nowadays is accepted to be ubiquitously expressed throughout the body [14]. In contrast, CB_2_ is expressed at high levels in leukocytes, neutrophils, keratinocytes, the spleen, natural killer cells, and, at a lower extent, in the muscle, liver, intestines and testes [40], as well as in the adipose tissue [41]. However, the second isoform of CB_2_ seems to be present in additional tissues, especially in the brain and kidney [40]. Although CB_1_ and CB_2_ are well known and characterized, numerous pharmacological studies suggest the existence of additional cannabinoid receptors. Recent data point to two other GPCRs, G protein-coupled receptor 55 (GPR55) and G protein-coupled receptor 119 (GPR119) as novel potential cannabinoid receptors (reviewed [42]), besides the transient receptor potential vanilloid type 1 (TRPV_1_) ion channel, which is well-known to bind some endocannabinoid ligands. The human orphans GPR55 and GPR119, originally identified through a bioinformatic approach [43], were both cloned in mice, rats and humans [44]. The human GPR55 shares only 14% sequence identity with the CB_1_ and CB_2_ receptors and is mainly expressed in the brain (caudate and putamen, cerebellum) [44,45]. Thus, GPR55 might be involved in learning, memory, and motor function given its high expression in the brain, especially the basal ganglia and cerebellum [44,45]. The human GPR119 is encoded by a protein of 335 amino acids, and isoforms of this receptor are present in various mammalian species [44]. Expression profiles of GPR119 mRNA receptor seem to be restricted to the pancreas, fetal liver and gastrointestinal tract in humans [46,47].

#### 1.1.4. Biological Effects of Cannabinoids via Their Receptors

**Figure 2:**
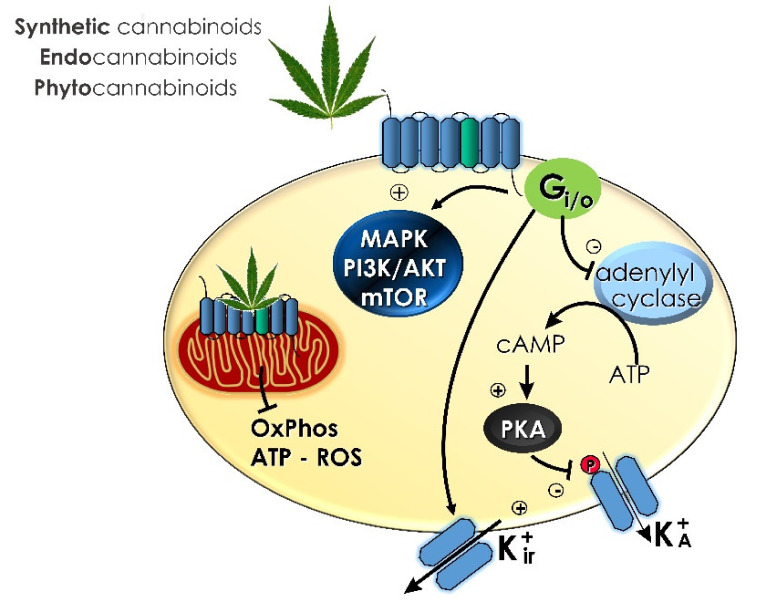
Summary of the signaling pathways associated with cannabinoid administration.

Cannabinoids exert their physiological and pathophysiological effects mainly by binding to various cannabinoid receptors and triggering different signaling pathways (Figure 2). Here, we will mainly focus on the best described amongst them, which is the CB_1_ receptor [48]. The central mechanism of action of CB_1,_ when activated, is to inhibit adenylate cyclase, a second messenger system, in a dose-dependent manner via Gi/o proteins, which reduce intracellular levels of cyclic adenosine monophosphate (cAMP) [49,50]. This turn results in a downregulated activity of cAMP-dependent protein kinase (PKA), which in turn reflects on downstream signaling pathways, such as ion channels, and electrical properties of the cell, triggering several mitogen-activated protein kinases (MAPK) [51].

Amongst other signaling pathways which have been shown to play a key role in the cellular and behavioral effects of THC PI3K/Akt signaling, the mTOR pathway and neurosteroid synthesis are worth mentioning (reviewed in [48]). Furthermore, a series of recent studies point out that apart from their canonical plasma-membrane localization and signaling, CB_1_ receptors are also associated with mitochondrial membranes in several cell types. Activation of these subcellular receptor pools tremendously impacts cell bioenergetic status, resulting in important behavioral and physiological alterations [52,53,54,55,56,57].

### 1.2. Oral and Craniofacial Cannabinoid Receptors

#### 1.2.1. Tongue

Several studies found the expression of both CB_1_ and CB_2_ receptors in the human tongue [58]. Immunohistochemical positive CB_1_ and CB_2_ immunoreactivity throughout the full thickness of the epithelium has been found in the epithelial cells of the tongue and in circumvallate and fungiform papillae [59]. Moreover, both CB_2_ and TRPV_1_ receptors have been described in epithelial cells adjacent to taste buds and in the basal layers of tongue epithelium [60,61,62].

However, how cannabinoids are involved in tongue functions is still unclear. To date, the elegant series of studies performed by Yoshida and colleagues showed that administration of both exogenous agonists and endogenous cannabinoids increases gustatory nerve responses to sweeteners, as well as behavioral responses to sweet–bitter mixtures, and electrophysiological responses of taste receptor cells to sweet compounds [60,61,63,64,65,66,67,68,69]. Interestingly, genetic and pharmacological receptor blockades highlight an exclusive role of CB_1_ receptors in the aforementioned cannabinoid effects [60,61]. The pathophysiological status of the tongue has been recently associated with cannabinoid receptor expression levels. Indeed, several pieces of evidence found a higher expression of both CB_1_ and CB_2_ receptors in patients suffering from mobile tongue squamous cell carcinoma (SCC) [70]. Moreover, higher levels of TRPV_1_ and CB_2_ are also associated with a reduction in CB_1_ expression levels, which have been described in the epithelial cells of the tongue from patients with burning mouth syndrome [59]. These last observations are in line with the role of cannabinoid receptors in cancer [71] and inflammation [72], which will be treated in the next session.

#### 1.2.2. Salivary Glands

**Figure 3:**
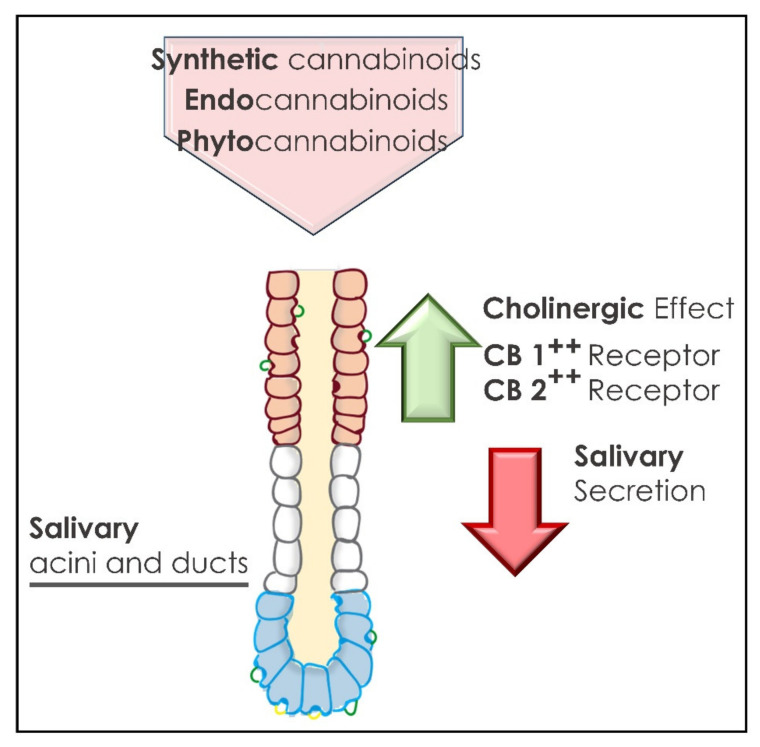
Salivary glands’ acini and ducts activity associated with cannabinoid administration.

Salivary glands express both CB_1_ and CB_2_ receptors with specific patterns [73,74,75,76,77,78,79,80,81,82,83,84,85,86]. CB_1_ receptors have been detected in the major salivary glands, however their expression was not observed in the acinous cells but were restricted to the striated duct cells near to the apical membrane [87]. CB_2_ receptors instead have been visualized mainly in myoepithelial cells surrounding the acini, where the production and release of saliva takes place, as well as in neurons of ganglia from the secretory ducts (Figure 3) [88]. Cannabinoid receptor expression in salivary glands has been shown to be under the control of several factors, including food quantity and quality and noradrenergic tone [74,88]. For instance, in the submandibular gland, basolateral membranes of ductal cells primarily express CB_1_ which, however, is also found in the serous cells of mixed acini according to dietary status [88]. Several pieces of evidence from the Elverdin lab pointed out a negative action of both CB_1_ and CB_2_ receptor activation in the regulation of saliva secretion [89,90,91,92,93], which might explain the dry mouth sensation always experienced by heavy cannabis users [11]. These sets of findings were supported by another study showing that endogenous cannabinoid anandamide, by activating CB_1_ receptors expressed in rat parotid glands, triggers cAMP accumulation. This results in amylase release with subsequent Na^+^ –K^+^–ATPase inhibition and impacts upon salivary gland functions [73].

#### 1.2.3. Pulp Tissue

Although in dental pulp tissues only few reports succeed in the detection of CB_1_ receptor expression, several reports pinpoint out a therapeutical role of cannabinoids in this oral tissue. Indeed, CB_1_ receptors have been found at the pulp–dentin border, especially located on the nerve terminals impinging into the dental pulp tissue, and this pattern of expression was maintained in nerve fibers of symptomatic painful dental pulp [94]. Given the well-known role of neurotransmitter suppressors in basically all kinds of transmission [95], together with the presence of CB_1_ receptors on these nerve terminals cannabinoids might represent a good therapeutic target for diseases with dental pain. Another target of cannabinoid-based medicine in the dental pulp might be dentin repair/regeneration. Indeed, functional CB_1_ receptors have also been reported in human odontoblasts [96]. Cannabinoid treatment of rat odontoblasts has been shown to promote the formation of “reparative dentin” by modulating extracellular Ca^2+^ entry [97], which might be the mechanism for CB_1_-mediated dental pulp tissue repair via the matrix metalloproteinase–2 activation in dental pulp cells [98,99,100,101,102].

#### 1.2.4. Periodontal Tissue

In periodontal tissues, several reports have suggested a role for both CB_1_ and CB_2_ receptors in pathological conditions, such as inflammation and wound healing [103,104,105]. Indeed, CB_1_ are expressed at a significantly higher level than CB_2_ receptors in both epithelium and periodontal ligaments (PDL) in periodontal tissues from healthy subjects. Furthermore, there is a switch in receptor expression (downregulation of CB_1_ and overexpression of CB_2_ receptor) within the PDL following bacterial inflammation. On the other hand, sterile inflammation strongly increases CB_1_ and CB_2_ expression in the PDL, but not in the alveolar bone nor in the cementum [103].

Periodontal tissue cannabinoid receptors have been suggested to differentially regulate cell growth and differentiation, inflammatory processes, and tissue healing [104,106,107,108,109,110,111,112,113,114,115], indicating that distinct expression patterns of CB_1_ and CB_2_ in PDL may be representative of distinct cellular function [104,106,107,108,109]. For instance, Liu et al. showed that cannabinoids, by activating FAK and MAPK signaling in a CB_2_-dependent manner, trigger periodontal cell adhesion and migration [104], which provides evidence for therapeutic potential of cannabinoid compounds in periodontal regeneration and wound healing, possibly associated with the anti-inflammatory actions of CB_1_ receptor activation, via NF-kappaB pathway inhibition in the periodontal tissue, as reported by Nakajima and colleagues [109].

#### 1.2.5. Oral Mucosa

At a histological level, oral mucosa is made by a stratified squamous epithelium and underlying connective tissues. Although no direct report on cannabinoid receptor expression in oral mucosa has yet been provided, CB_1_ and CB_2_ have been shown to be functionally expressed by skin epithelial cells, suggesting a putative role in modulating several cellular functions in the mucosa epithelium [116]. Indeed CB_1_ and CB_2_ receptor activation exerts opposite effects on human epidermal keratinocyte proliferation and differentiation [117,118,119,120,121,122,123,124,125,126,127]. As previously mentioned, CB_1_, CB_2_ and TRPV_1_ receptors are indeed identified in the connective tissue from the lamina propria layer from the oral mucosa especially on salivary glands, blood vessels, nerve endings, and immune cells belonging to this tissue [59]. However, there is to date a poor scientific description of cannabinoid receptor expression in the oral mucosa, an issue that will need to be addressed since oral mucosa is the first line of tissue interacting with cannabinoids during marijuana consumption. Thus, exploring the physiological and pathophysiological role of cannabinoids on oral mucosal health and diseases might represent the way to improve cannabis-based medicine or mitigate side effects of cannabis recreational consumption. The aim of the present investigation was to evaluate the cannabinoids and their biological effects through a systematic review of the literature.

## 2. Materials and Methods

### 2.1. Patient and Public Involvement

The present investigation evaluated the effects of cannabinoids on oral health associated with recreational using and therapeutic purposes through a systematic review of the literature.

No patients have been involved in the present study, while no investigational ethical considerations are associated with the present paper.

### 2.2. Search Strategy

The study PICO question has been summarized in Table 1, and the scope of the present investigation was to evaluate the effectiveness of cannabinoids derived adjuvant for the treatment of different diseases of the oral cavity such as: dry mouth, tooth caries, periodontal and gingival diseases, oral hygiene maintenance, oral cancer and oral tissue diseases.

The paper search and selection was conducted independently by two expert reviewers (F.I. and F.L.), and a Boolean database search has been conducted in the Pubmed (MEDLINE) and EMBASE electronic databases without any time limitations. The key words search indicators are presented in Table 2: (cannabinoids AND dry mouth); (cannabinoids AND caries); (cannabinoids AND periodontal diseases); (cannabinoids AND oral hygiene); (cannabinoids AND oral cancer); (cannabinoids AND oral tissue diseases). Moreover, a manual paper search was conducted to improve the article pool; the duplicates were removed after the title evaluation. The abstracts were manually evaluated to perform an initial screening of the articles identified and the final selection was performed with the full text of the papers in order to conduct the eligibility for the qualitative analysis. At the end of the process, the papers selected were categorized according to the reference data, year of publication, type of the study, patients treated, test and control group treatments, follow-up, and study effectiveness.

### 2.3. Inclusion and Exclusion Criteria

For the present investigation, for the qualitative analysis full-length articles written in English language were considered, as well as literature reviews and meta-analyses, randomized and non-randomized clinical trials, case reports and case series. The exclusion criteria for the evaluations were: editorial letters, book chapters and conference proceedings.

### 2.4. Study Selection

The full texts were recorded and evaluated for all the papers included in the present systematic review. Each one was studied independently according to the inclusion and exclusion criteria mentioned above. The majority of the papers were in the English language; we only choose the ones in which the drilling technique was performed following the guidelines of the burst producer. The minimum follow up period was set to three weeks.

### 2.5. Data Extraction

For the qualitative synthesis of the studies included, the following data were considered: the drug description, the design of the study, the experimental model, the administration protocol, and the effectiveness of the study.

## 3. Results and Discussion

### 3.1. Articles Selection Process

**Figure 4:**
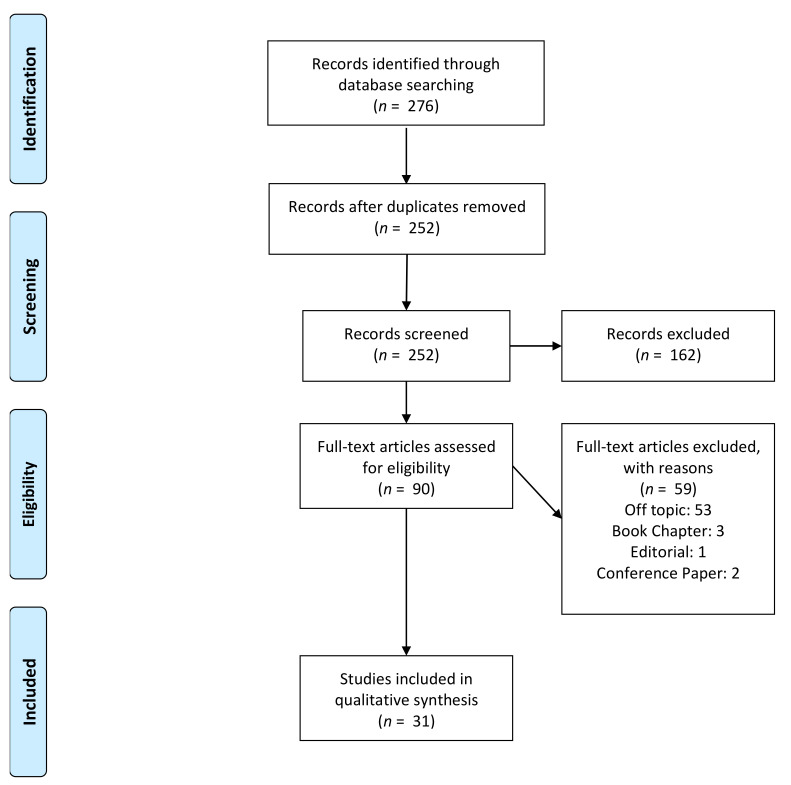
PRISMA flowchart of the article screening and inclusion for the qualitative synthesis [128].

The entire article identification, initial screening, eligibility assessment criteria and qualitative analysis processes are described in Figure 4. The initial screening process retrieved a total of 276 articles. The papers identified were merged, and after the initial screening a total of 162 articles were excluded. The eligibility assessment was performed and a total of 59 manuscripts were excluded from the articles pool: 53 off topic papers, 3 book chapters, 1 editorial letter and 2 congress proceedings. A total of 31 articles were selected for the qualitative synthesis.

### 3.2. Cannabinoids Drugs for the Treatment of Dry Mouth

A total of four studies were included about cannabinoid use and dry mouth disease. Darling et al. reported the only cross-sectional study conducted on 300 patients that reported cannabinoids consumption by smoking (Table 3) [129]. The subjects included reported nicotinic stomatitis in a total of four cannabis consumers but not smokers. A higher incidence of leukoedema and dry mouth was evident in cannabis users compared to the control groups. The other studies were conducted on animals: two papers on rat models [89,92] and one article on pigs [88]. Pirino et al. evaluated the cannabinoid receptor expressions CB_1_ and CB_2_ after a dietary supplement administration on 32 pigs, reporting an influence of the expression of salivary ducts and secretion of the mandibular glands related to endocannabinoids activity (Table 3).

### 3.3. Cannabinoids and Dental Caries

A total of three studies were included about the topic of cannabinoids and dental caries. Two articles reported a clinical study on humans: a case report [130] and a retrospective cohort trial [131]. Grafton et al. [130] reported a clinical report of a low compliance of a marijuana smoker that submitted to a tooth extraction procedure with a high incidence of dental caries. Ditmyer et al. [131] reported through a retrospective cohort study on 66,941 subjects an increase of the prevalence and severity of dental caries in patients that declared tobacco/marijuana administration. In vitro, Liu et al. [104] reported that delta-9-tetrahydrocannabinol (THC) promoted periodontal cell adhesion and migration in wound tissue healing (Table 4).

### 3.4. Cannabinoids and Periodontal Diseases

A total of 10 articles were included about the topic of cannabinoids and periodontal diseases: two clinical studies, three studies only in vitro, one study only in vivo on rats and three articles with both in vitro/in vivo on rats. Thomson et al. [132] reported in patients affected by periodontitis that the cannabis smoking may be a risk factor for periodontal disease independent from the tobacco use, while Shariff et al. [133] showed that cannabis smoking was correlated to deeper probing depths, increased clinical attachment loss and higher risk for severe periodontitis. Nogueira-Filho et al. [134] reported on rats that cannabis smoke exposure may impact alveolar bones by increasing bone loss, while in other studies the administration of synthetic cannabinoid derived molecules such as anandamide (AEA)/2-arachidonoylglycerol (2-AG)+ AM251, AM630 and HU-308 seems to be correlated with an increased activity and proliferation of human gingival fibroblasts, a lower bone loss by the inhibition of the RANK/RANKL expression, and anti-inflammatory and osteoprotective effects on the oral tissue in vivo [107,135,136,137]. In studies conducted on human periodontal fibroblasts (HPLF) and human gingival fibroblasts, the cannabinoids exhibited a strong inhibition of pro-inflammatory molecules such as LPS, TNF-α, and IL-1β expression [106,107,108] (Table 5).

### 3.5. Cannabinoids and Oral/Neck Cancer

A total of 13 articles were included about the topic of cannabinoids and oral/neck cancer development: three literature reviews [138,139,140], four in vitro studies [141,142,143,144], one case series, and five case–control and cohort studies [145,146,147,148,149]. The studies [138,145,147,148,149] that evaluated marijuana consumption reported that the smoking habitude has been correlated to a carcinogen induction with no completely clarified chemical and physical pathogenesis, while Rosenblatt et al. [146] demonstrated a similar oral cancer incidence between test and control with no cannabis smoke evidence. The studies [141,142,143,144] that considered cannabinoids supplements in vitro reported a capability to inhibit the growth of different cancer cells lineages, including aggressive and chemotherapy-resistant variants of lung cancers (Table 6).

### 3.6. Cannabis and Oral Tissue Diseases

A total of two studies were included for the qualitative synthesis: a literature review [11] and a cross-sectional study on humans [129]. Versteeg et al. [11] reported that the cannabis smoking habit has been correlated with an increased incidence of xerostomia, leukoedema and a higher prevalence of Candida albicans infections. Darling et al. [129] reported a high incidence of nicotinic stomatitis associated with cannabis consumers with no tobacco use.

### 3.7. Cannabis Consumption and Effect on Oral Health

**Figure 5:**
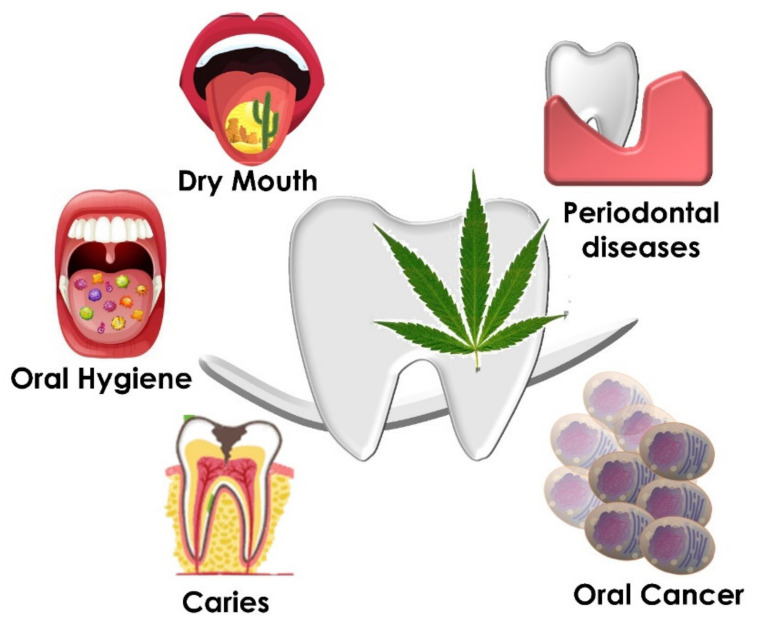
Oral pathologies and disease involved with cannabinoid exposure and abuse.

Cannabis abuse has always been known to impact on proper oral health status. Several compounds assume that cannabis smoke will possibly put cannabis users to a higher risk of dry mouth, dental caries, soft tissue disease, poor oral hygiene, periodontal disease and even oral cancer by changing the physiology of the oral environment (Figure 5). On the other hand, cannabis might represent a good pain management tool for dental anesthesia as well as post-operative management.

### 3.8. Dry Mouth

Cannabis use can lead to xerostomia by reducing salivary flow. Dry mouth associated with cannabis abuse is reported to be similar to the one after cigarette smoking, and in most subjects dry mouths appear immediately after cannabis use [129]. Cannabis use has always been associated with dry mouth and hypo-salivation via a CB_1_/CB_2_ receptor-mediated THC effect on the salivary glands cholinergic transmission [89,92]. THC has also been shown to importantly reduce submandibular salivary flow induced by electrical stimulation in dogs [150]. These findings may help to better understand the mechanisms of reduced saliva production, which eventually lead cannabis smokers to xerostomia.

### 3.9. Caries

Amongst the main dental complication of cannabis use, an increased incidence of caries has frequently been reported. This is probably mediated by several factors, which might include less saliva production, poor oral hygiene and higher plaque scores. Indeed, cannabis smokers have been shown to present a higher number of DMF teeth scores with a greater accumulation of plaque [130]. Another study, after correcting some confounding factors such as exposure to second-hand smoke, gender and race/ethnicity, reported an increased prevalence and severity of dental caries among marijuana users [131]. However, one has to also take into account the potential beneficial roles of cannabinoids on dental pulp diseases and regeneration/repair [104,106,107,108,109], which will be discussed in the next section.

### 3.10. Periodontal Diseases

To date, a potential link between cannabis use and periodontal disease is supported only by a limited and inconsistent literature background. Some studies tend to suggest chronic cannabis use as a potential risk factor for periodontal diseases including gingival leukoplakia, gingival hyperplasia, alveolar bone loss and gingivitis [132]. Additionally, a US Survey supports an incidence of more severe periodontitis associated with recreational cannabis use [133]. Higher bone loss and lower bone density were associated with marijuana smoke inhalation (MSI) in rats following ligature-induced periodontitis [134] with, however, no significant histological differences.

On the other hand, no association between cannabis smoking and periodontitis was found in another groups of studies. For example, no significant associations between cannabis use and periodontitis have been found in adolescent populations [151]. Moreover, in mice with ligature-induced periodontitis, cannabinoids have been shown to protect them from periodontal diseases, as CBD/THC injection strongly reduced pro-inflammatory cytokine levels and PMN cell motility as well as less furcation bone loss [137].

Several pieces of evidence against the causative effects of cannabinoids on periodontal disease are given by the well-known role of the endocannabinoid system in periodontal healing, as mentioned previously. Cannabinoids, by activating CB_1_/CB_2_ receptors, promote the proliferation of gingival fibroblasts in periodontal healing [107], and methanandamide and HU308, selective CB_1_ and CB_2_ receptor agonists, are able to dampen LPS-induced periodontitis in vitro and in vivo [135,136], especially by attenuating alveolar bone loss and increased inflammatory mediator. Moreover, administration of CBD inhibited RANK/RANKL expression resulting in a diminished bone resorption and pro-inflammatory cytokine in the periodontal tissue [137]. Thus, these findings highlight different receptor and molecular mechanisms on periodontal disease, which are all in support of an anti-inflammatory and protective effects of cannabinoids.

Multiple factors and research designs might explain the conflicting findings for the link between cannabis use and periodontal disease. First, patients presented several risk factors apart from cannabis use such as age, systemic health, concurrent tobacco smoking and oral hygiene. Second, individuals had different amounts, frequencies, duration, and modes of administration of cannabis use. Third, the effects of cannabis use on oral tissues and oral health have been described only in limited reports; thus, more well-designed studies will be needed to address these issues.

### 3.11. Oral Hygiene

Cannabis abusers, as well as cigarette smokers, normally have poor oral hygiene and higher plaque scores, increasing the likelihood of caries and periodontal disease [152]. Unfortunately, it is difficult to determine whether neglect of oral hygiene and failure to seek regular preventative dental care might be the causes directly linking cannabis use to oral uncleanliness. One study showed that increasing amounts of drug used was not associated with a lower oral hygiene index, or decayed, missing and filled teeth (DMF–T) [129]. As cannabis users often also abuse tobacco and alcohol, this relationship is of course hard to disentangle.

### 3.12. Oral Cancer

Although still unclear, an association between marijuana use and oral cancer has been recently proposed. Indeed, cannabis smoke increases the possibility of developing oral cancer, since it contains similar carcinogens as in tobacco. Some studies indicate that cannabis use increases oral premalignant lesions such as leukoplakia and erythroplakia, especially on the anterior floor of the mouth and the tongue [129,138]. Cannabis smoking has also been suggested to be a possible cause of tongue carcinoma [138,145,146], and marijuana smokers have been found with epithelial dysplasia in the buccal mucosa [129]. A strong association between cannabis use and head and neck cancer has also been reported among younger patients [145,147]. Furthermore, frequent, forever and long duration marijuana use increases significantly the possibility of developing oropharyngeal cancer [147].

However, other studies failed to associate cannabis use to head and neck cancer [139,148,149,153]. Moreover, a case-control study with strict control for confounding factors, such as birth year, education, sex, cigarette and alcohol consumption, showed no association between oral squamous cell carcinoma before and after cannabis consumption [146], indicating that conflicting results may be due to different methods used, and a lack of quality research. Targeting the cannabinoid system represents a potential therapeutic target in the treatment of several types of cancer [140]. Cannabinoid agonists prevent cancer cell progression, reducing tumor growth and metastasis in at least in two ways: by inhibiting cancer cell proliferation and/or inducing autophagy and cell apoptosis [143,144] by suppressing cancer cell migration [141,142]. Thus, the potential of therapeutic targeting of cannabinoid receptors in oral cancers should not be neglected.

### 3.13. Other Oral Tissue Diseases

Cannabis smoking may also result in lesions in the oral soft tissue. Stomatitis with leukoedema and hyperkeratosis are often found in the buccal mucosa of cannabis smokers, probably resulting from the high temperature of the smoke or the specific chemicals inhaled [129]. Moreover, due to their poor oral/denture hygiene and nutritional deficiency, heavy cannabis users are also more prone to *Candida albicans* infections [11].

### 3.14. Potential Therapeutic Application of Cannabinoids on Oral Health

As mentioned before, its anti–oxidant, anti–inflammatory and analgesic properties have allowed CBD to be proposed as a therapeutic and safe drug for use in oral mucositis [154], Thus, this recent proposition of CBD use in dentistry will surely open the way to studies on the use of cannabinoids in oral mucositis and other oral mucosal diseases caused by oxidative stress, chemotherapy, or radiotherapy.

There are many considerations of the role of marijuana’s effect with dental anesthesia, especially as a pain management tool for surgical analgesia as well as post-operative management. In a study done by Holdcroft et al., capsules of THC and CBD were given to patients following major operations [155]. Pain relief and mood, measured by eight assessments trough a visual scale, showed that these capsules reduced demands and extended the lag time for rescue analgesia (morphine) in patients; the optimal dosage, to avoid dose-related side effects such as dizziness and sedation, was ten milligram [155]. This and other studies showed morphine-sparing effects of cannabis, which are crucial as opioid compounds have high abuse potential and fatal risks [156], indicating the potential use of marijuana as an analgesic alternative with positive future implications for the dental field.

## 4. Conclusions

Although there is a long history of cannabis use, the knowledge of the effects of cannabis on human health has only been enriched in recent decades. The discovery of synthetic cannabinoids, cannabinoid receptors and the endocannabinoid system has paved the way for better understanding of several effects of cannabis on the human brain and body. Given the present and future increase in health issues related to recently legalized cannabinoid consumption, it is mandatory for oral healthcare providers and dentists to know and understand both the adverse and beneficial oral effects of cannabis. It is critical for oral healthcare providers to be aware of a patient’s status, to recognize the potential risks, and to seek the best treatment options.

The most common way of consuming cannabis, marijuana smoking, has several direct and indirect deleterious effects on oral cavities; however, the evidence linking cannabis to oral/dental diseases is contradictory and at best limited. This is often related to different personal risk factors, as well as the lack of details in marijuana usage information.

Innovative compounds active on selective cannabinoids receptors could be useful for the treatment of numerous systemic disease and novel implications in several pathologies.

Well-designed research controlling for confounding factors are needed in the future, and more basic and clinical research should be designed to understand the mechanisms of action of cannabis. This will allow us to precisely target the systemic and oral effects in a more specific manner, by developing synthetic agonists, antagonists and more general modulators of the endocannabinoid system. This will largely benefit patients by developing new therapeutic approaches to increase treatment efficacy and to reduce the side effects.

## Figures and Tables

**Table 1:** PICO questions explication.

PICO			
Population\Patients	Intervention	Comparison	Outcomes
Patient group of interest?	What is the main intervention you wish to consider?	Is there an alternative intervention to compare?	What is the clinical outcome?
Patients that need treatment for dry mouth/caries/periodontal diseases/oral hygiene/oral cancer/oral tissue diseases	Treatment protocol with cannabinoids derived adjuvants	Treatment protocol without cannabinoids derived adjuvants	Can this cannabinoid derived adjuvant provide an higher effectiveness for dry mouth/caries/periodontal diseases/oral hygiene/oral cancer/oral tissue diseases

**Table 2:** Electronic database Boolean search: keyword strategy.

	Search Strategies
Keywords:	Advanced search: (cannabinoids AND dry mouth); (cannabinoids AND caries); (cannabinoids AND periodontal diseases); (cannabinoids AND oral hygiene); (cannabinoids AND oral cancer); (cannabinoids AND oral tissue diseases);
Databases	PubMed/Medline, EMBASE

**Table 3:** Summary of the studies included according to the cannabinoids and dry mouth.

Cannabinoids and Dry Mouth									
Authors	Drug	Study Design	Experimental Model	Administration Protocol	Results	Test	Control	Subjects/Specimens	Study Time
Darling et al. [129]	smoke	Cross-sectional study	oral tissues health and oral dryness was measured.	-	nicotinic stomatitiswas reported in four cannabis consumers not tobacco users, Leukoedema and dry mouth was more evident in cannabis users	cannabis/tobacco/methaqualone smokers	Control 1:152 tobacco; Control 2:189 non-smokers	300 subjects	-
Pirino et al. [88]	Dietary supplements	In vivo on pigs	Pigs Mandibular glands cannabinoid receptors type 1 (CB_1_) and cannabinoid receptors type 2 (CB_2_) expression	Dietary supplements administration	endocannabinoids may influence the functional activity of the mandibular gland modifying qualitative and/ or quantitative activity and CB_1_ CB_2_ receptors expression of salivary duct and secretion.	finely ground pellet (FP), coarsely ground meal (CM), coarsely ground pellet (CP) and coarsely ground extruded (CE)	-	32 samples	4 weeks
Prestifilippo et al. [89]	Right femoral vein administration	In vitro study/In vivo on rats	Salivary glands histological evaluation/Ducts cell gene expression	In vivo Salivary Secretion evaluation. In vitro: genes expressions	AEA decreases saliva secretion in the SMG-acting through CB_1_ and CB_2_ receptors.	anandamide (AEA), forskolin (FRSK), NE-HCl, Chloralose and methacholine (MC)	No treatment	40 samples	3 min, 10 min
Prestifilippo et al. [92]	Systemic administration/Intraduct salivary gland administration	In vitro study/In vivo on rats	Salivary glands histological evaluation/Ducts cell gene expression in the presence of inflammogens (LPS)	In vivo Salivary Secretion evaluation. In vitro: genes expressions	endocannabinoids mediate the hyposialia induced by inflammogens in the SMG and in the brain.	LPS and/or the cannabinoid receptor antagonist AM251 administration	cannabinoidreceptor antagonist AM251 administration		

**Table 4:** Summary of the studies included according to the cannabinoids and caries lesions.

Cannabinoids and Caries Lesions									
Authors	Drug	Study Design	Experimental Model	Administration Protocol	Results	Test	Control	Subjects/Specimens	Study Time
Grafton et al. [130]	Marijuana/Tobacco Smoke	Case Report	Tooth extraction socket/Dental Caries	5 h before the dental treatment	Low patient compliance regarding the cannabis use.	-	-	1 subject (29 years old)	-
Ditmyer et al. [131]	Marijuana/Tobacco Smoke	Retrospective cohort study	Dental Caries Prevalence Screening		High prevalence/severity of dental caries in subjects with tobacco/marijuana administration			66,941 subjects (13–18 years old)	8 years
Liu et al. [104]	Tetrahydrocannabinol (THC)	In vitro study	Human Periodontal fibroblast (HPLF)	Cell cultures	THC promoted periodontal cell adhesion and migration through wound healing	THC 1µM	No treatment	-	0 h, 3 h, 6 h and 24 h

**Table 5:** Summary of the studies included according to the cannabinoids and periodontal lesions.

Cannabinoids and Periodontal Lesions									
Authors	Drug	Study Design	Experimental Model	Administration Protocol	Results	Test	Control	Subjects/Specimens	Study Time
Kozono et al. [107]	Endocannabinoid	In vitro study/In vivo on rats	Periodontal fibroblasts/periodontal wound healing	Cell culture	Higher proliferation of human gingival fibroblasts (HGFs) by AEA, that can be reduced by AM251 and AM630, selective antagonists of CB_1_ and CB_2_	anandamide (AEA)/2-arachidonoylglycerol (2-AG)	anandamide (AEA)/2-arachidonoylglycerol (2-AG)+ AM251 and AM630, which are selective antagonists of CB_1_ and CB_2_,	4 specimens	0, 3 days, 7 days, 14 days
Thomson et al. [132]	Cannabis Smoking	Prospective cohort study	Periodontitis	Cannabis exposure	Cannabis smoking may be a risk factor for periodontal disease that is independent of the use of tobacco	1: cannabis some exposure; 2: cannabis high exposure (182; 20.2%).	No exposure	1037 subjects	1 year
Shariff et al. [133]	cannabis (marijuana and hashish)	Cohort study	Periodontal examination	-	Cannabis use was related to with deeper probing depths, more clinical attachment loss and higher odds of having severe periodontitis.	Cannabis exposure	Non cannabis users	1938 subjects	1 year
Nogueira-Filho et al. [134]	Cannabinoids	In vivo on rats	Experimental periodontitis	Cannabis exposure	cannabis smoke may impact alveolar bone by increasing bone loss	marijuana smoke inhalation	No exposure	30 specimens	30 days
Ossola et al. [135]	synthetic cannabinoid	In vitro study/In vivo on rats	Lipopolysaccharide-Induced Periodontitis	topical administration on gingival tissues	anti-inflammatory, osteoprotective and pro-homeostatic effects of HU-308 in oral tissues	1: Vehicle; 2: HU-308 (500 ng/mL); 2: LPS/HU-308 (500 ng/mL)	No treatment	24 specimens	45 days
Napimoga et al. [137]	Cannabis Smoking	In vivo on rats	LPS Experimental periodontitis	Vein administration	Cannabidiol is related to a lower bone resorption by the inhibition of the RANK/RANKL expression	1: vehicle; 2: Cannabidiol (CBD)	No treatment	30 specimens	30 days
Ossola et al. [136]	synthetic cannabinoid	In vitro study/In vivo on rats	Lipopolysaccharide-Induced Periodontitis	topical Meth-AEA (500 ng/mL)	Beneficial effects of treatment with Meth-AEA on gingival tissue of rats with periodontitis.	1: synthetic cannabinoid methanandamide (Meth-AEA); 2: LPS/(Meth-AEA); 3: LPS	No treatment	24 specimens	6 weeks
Abidia et al. [106]	Cannabinoid	In vitro study	Human Periodontal fibroblast (HPLF)	cannabinoid compounds (10^−4^–10^−6.5^ Min cell culture	The cannabinoids inhibited LPS, TNF-α, IL-1β expression in hPDLFs though CB2R ligands receptors	cannabinoid (10^−4^–10^−6.5^ M) [EC_50_]		-	1 h
Lanza Cariccio et al. [108]	Endocannabinoid	In vitro study	Periodontal fibroblasts	Cells culture	Higher survival capacity and neuronal differentiation potential of hPDLSCs treated with Moringin and Cannabidiol	Moringin (MOR) and Cannabidiol (CBD),	No treatment	-	24 h, 48 h and 72 h
Nakajima et al. [107]	Endocannabinoid	In vitro study	human gingival fibroblasts (HGFs)	Cells culture	AEA blocked of LPS-triggered NF-jB activation related to hyperinflammatory response in periodontitis.	Anandamide (AEA)/LPS in different concentrations (0, 1µM, 5µM and 10 µM)	-	-	48 h

**Table 6:** Summary of the studies included according to the cannabinoids and oral and neck cancer.

Cannabinoids and Oral and Neck Cancer									
Authors	Drug	Study Design	Experimental Model	Administration Protocol	Results	Test	Control	Subjects/Specimens	Study Time
Firth et al.	Marijuana consumption	Literature review	Case report literature overview	Smoking aptitude	The marijuana mechanisms related to the carcinogen are not clearly clarified and probably related to, aromatic hydrocarbons, benzo[a]pyrene and nitrosamines in smoked cannabis	Cannabis consumption/two cases in combination with heavy tobacco use		8 subjects	-
Donald et al.	Marijuana consumption	Case series	Clinical reports	Smoking aptitude	The active euphoria-producing agent, 1-9 tetrahydrocannabinol, has been implicated In altered DNA, RNA, and protein synthesis and consequent chromosomal aberrations	Cannabis consumption/one cases in combination with heavy tobacco use	-	6 patients	-
Rosenblatt et al.	Marijuana consumption	case–control study	Young adult population	Smoking aptitude on a large population sample	A similar proportion of case subjects (25.6%) and control subjects (24.4%) reported ever the use of marijuana	Cannabis consumption	No tobacco use and no cannabis consumption	1022 subjects	-
Marks et al.	Marijuana consumption	Epidemiological study	INHANCE consortium USA and Latino-America database	Smoking aptitude on a large population sample	The associations of marijuana use with oropharyngeal and oral tongue cancer are consistent with both possible pro- and anticarcinogenic effects of cannabinoids	marijuana smokers	Nonsmokers	9916 subjects	
Hashibe et al.	Marijuana consumption	Cohort study	high school students and young adults population	Smoking aptitude	marijuana use was not associated with increased risk of all cancers or smoking-related cancers.	marijuana smokers	Nonsmokers	64,855 subjects	8 years
Llewellyna et al.	Marijuana consumption	Cohort study	Young adults <45 years old	Smoking aptitude	the major risk factor for oral cancer was consumption of alcohol or both. No evidence about marijuana consumption or tobacco	Multifactorial carcinogenic and diet quality analysis	-	116 subjects	7 years
Llewellyna et al.	Marijuana consumption	Case control study	Identification of the majorrisk factors for oral cancer in young adults	-	fresh fruits and vegetables in the diet appeared to be protective for both males and females. No evidence about marijuana consumption.	Multifactorial carcinogenic and diet quality analysis	-		7 years
Osazuwa-Peters et al.		Literature review	Identification of the co-relationship between cannabis consumption and oral cancer	Smoking aptitude	Insufficient evidence about the association between head and neck cancer and marijuana use	marijuana smokers	Nonsmokers	-	-
Guzman et al.	cannabinoids Supplements	Literature review	The cannabinoid derivate as an anticancer agent	-	Cannabinoids exert palliative effects in patients with cancer and inhibit tumor growth in laboratory animals.	Cannabinoids in combination with chemotherapeutic drugs or radiotherapy	-	-	-
Nabissi et al.	cannabinoids Supplements	In vitro study	multiplemyeloma cells	Cannabinoids/carfilzomib administration	The Δ9-tetrahydrocannabinol (THC)/cannabidiol (CBD) combination showed strong anti-myeloma activities.	Δ9-tetrahydrocannabinol (THC)/Cannabidiol (CBD)	-		72 h
Salazar et al.	cannabinoids Supplements	In vitro study	human glioma cells	Cannabinoids administration	THC can promote the autophagic death of human and mouse cancer cells	Δ9-tetrahydrocannabinol (THC)	-	-	10 days
Grimaldi et al.	cannabinoids Supplements	In vitro study	breast cancer cells	Cannabinoids administration	The cannabinoids showed a slowed down growth of breast carcinoma and inhibited its metastatic diffusion	Anandamide (AEA)	Control no treatment	-	21 days
Preet et al.	cannabinoids Supplements	In vitro study	lung cancer cell/in vivo on mice	Cannabinoids administration	therapeutic use of THC for the treatment of aggressive and chemotherapy-resistant variants of lung cancers.	Δ9-tetrahydrocannabinol (THC)		6 samples	21 days

## Data Availability

All experimental data to support the findings of this study are available contacting the corresponding author upon request.
